# Supplementary material for: Flavin Adenine Dinucleotide (FAD) and Pyridoxal 5′-Phosphate (PLP) Bind to Sox9 and Alter the Expression of Key Pancreatic Progenitor Transcription Factors
Source: Int J Mol Sci. 2022 Nov 14;23(22):14051. doi: 10.3390/ijms232214051 (PMC9694089; doi:10.3390/ijms232214051)
Supplement: Supplementary file 1 [file ijms-23-14051-s001.zip › ijms-2007661-supplementary.pdf]

SUPPLEMENTARY INFORMATION

Flavin Adenine Dinucleotide (FAD) and Pyridoxal 5′-Phosphate (PLP) Bind to Sox9 and Alter the Expression of Key Pancreatic Progenitor Transcription Factors

Zeyaul Islam [1], Noura Aldous [1,2], Sunkyu Choi [3], Frank Schmidt [3], Borbala Mifsud [2], Essam M. Abdelalim [1,2] and Prasanna R. Kolatkar [1,2,*]

[1] Diabetes Research Center (DRC), Qatar Biomedical Research Institute (QBRI), Hamad Bin Khalifa University (HBKU), Qatar Foundation, Doha P.O. Box 34110, Qatar
[2] College of Health and Life Sciences, Hamad Bin Khalifa University (HBKU), Qatar Foundation, Doha P.O. Box 34110, Qatar
[3] Weill-Cornell Medical College in Qatar, Ar-Rayyan P.O. Box 24144, Qatar
* Correspondence: pkolatkar@hbku.edu.qa; Tel.: +974-445-45889; Fax: +974-445-41770

Supplementary Figure S1.

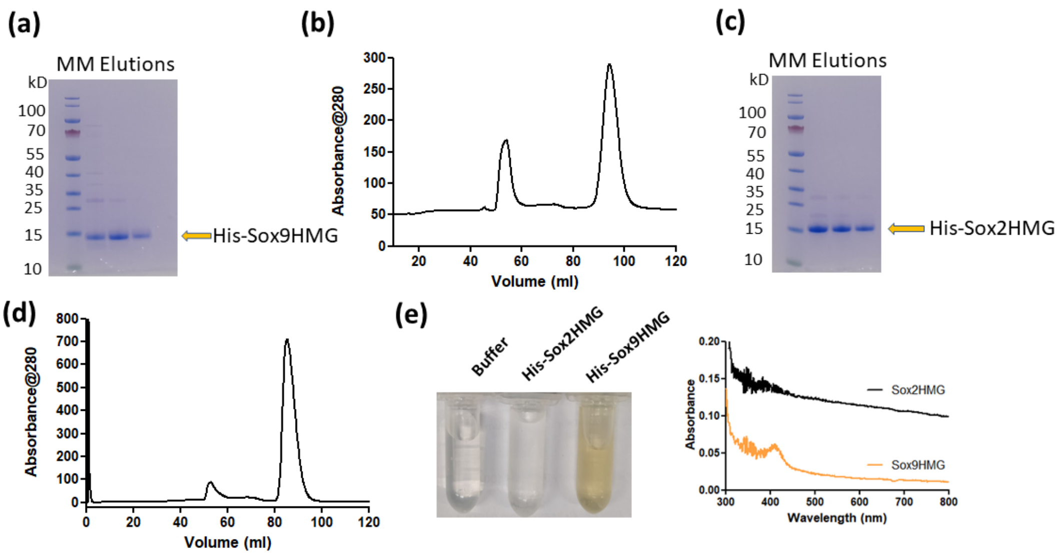

Supp. Figure S1: Purification and Uv-Vis spectroscopic characterization of Sox9 HMG and Sox2 HMG. (a) Sox9 HMG was purified using affinity chromatography exploiting the 6xHis tag at the N-terminal of the protein. (b) Protein was further purified using gel filtration chromatography (Superdex S75 column). (c) Sox2 HMG was purified using affinity chromatography exploiting the 6xHis tag at the N-terminal. SDS-PAGE of the eluted fractions corresponding to Sox2 HMG construct. (d) Sox2 HMG was further purified using gel filtration chromatography (Superdex S75 column), showing a homogenous and monodisperse peak. (e) Uv-Vis spectroscopic spectra of the purified Sox2 HMG and Sox9 HMG. Buffer was used as a reference
